# Nicotine protects fetus against LPS-induced fetal growth restriction through ameliorating placental inflammation and vascular development in late pregnancy in rats*

**DOI:** 10.1042/BSR20190386

**Published:** 2019-07-02

**Authors:** Junjie Bao, Yong Zou, Yuanyuan Liu, Li Yuan, Robert E. Garfield, Huishu Liu

**Affiliations:** 1 Department of Obstetrics, Preterm Birth Prevention and Treatment Research Unit, Guangzhou Women and Children’s Medical Center, Guangzhou Medical University, Guangzhou, China; 2 Department of Blood Transfusion, The Third Affiliated Hospital of Sun Yat-Sen University, Guangzhou, China; 3 Department of Pathology, Guangzhou Women and Children’s Medical Center, Guangzhou Medical University, Guangzhou, China; 4 Department of Obstetrics, The Affiliated Ganzhou Hospital of Nanchang University, Ganzhou, China

**Keywords:** fetal growth restriction, lipopolysaccharides, nicotine, pregnancy

## Abstract

Our previous work has shown that nicotine suppressed lipopolysaccharide (LPS)-induced placental inflammation by inhibiting cytokine release as well as infiltration of leukocytes into the placenta through the cholinergic anti-inflammatory pathway. Nicotine also increased fetal survival and restored pup weight. In the present study, we aim to further investigate if fetal growth restriction (FGR) occurs with LPS treatment, and evaluate the protective effects of nicotine on fetuses in late gestation of rats. Pregnant Sprague–Dawley rats were divided into control group, nicotine group, LPS group and LPS + nicotine group. Rats were first pretreated with nicotine or vehicle by subcutaneous injection on gestation day (GD)14 and GD15, followed by LPS or vehicle intraperitoneal injection on GD16, and were killed on GD18. Loss of fetuses, number and weights of live fetuses and weights of placentas were recorded. Placentas were collected to evaluate placental pathology and determine inflammatory cytokines and vascular endothelial growth factor (VEGF) levels. We found that LPS treatment increased levels of placental inflammatory cytokines and placental pathological damage, decreased levels of VEGF, reduced number of live fetuses and induced FGR. Pretreatment with nicotine reversed LPS-induced high levels of placental inflammatory cytokines, low levels of placental VEGF and placental pathological damage, then rescued the number and weights of live fetuses. These data demonstrated that activation of the cholinergic anti-inflammatory pathway by nicotine protected fetus against LPS-induced FGR through ameliorating placental inflammation and vascular development in late pregnancy in rats. It may be an alternative therapeutic strategy for inflammation- induced FGR in late pregnancy.

## Introduction

Fetal growth restriction (FGR) is the failure of a fetus to reach its full genetic growth potential. It is defined as fetal weight <10th percentile of a given population at the same gestational age. It affects 5–10% of pregnancies and is associated with increased preterm birth, low Apgar scores, hypoxemia, acidosis at birth, sepsis, intracranial hemorrhage, necrotizing enterocolitis, respiratory complications, fetal and neonatal death [1]. It affects the person throughout life course and is associated with a higher risk of developmental impairments including cognitive development, medical and health outcomes in adulthood [2–4].

The primary cause of FGR, when not attributable to structural or genetic defects of the fetus, is ‘placental insufficiency’ [5–7]. Although many environmental, nutritional, and hormonal factors are involved in anatomic and functional placental insufficiency, infectious or immunologic inflammation are also important contributors [8–11].

Recently, the cholinergic or nicotinic anti-inflammatory pathway has emerged as a system that contributes to regulation of inflammation [12,13]. The key features of this pathway are acetylcholine from the nervous system, principally the vagus nerve or other non-neural systems, interacts with a specific nicotinic receptor (α7 nicotinic acetylcholine receptor or α7-nAChR) to inhibit release of cytokines from cells, and thereby, exert systemic anti-inflammatory role [14]. The α7-nAChR agonists including nicotine show anti-inflammatory effects during experimental endotoxemia in rodents by suppressing pro-inflammatory cytokine release and leukocyte migration and recruitment [13]. Application of α7-nAChR agonists show that nicotine and other cholinergic agonists may be important regulators of inflammation in several experimental models of diseases, including autoimmune arthritis, sepsis, multiple sclerosis and ischemia–reperfusion injuries [15–18].

Previously, we used lipopolysaccharide (LPS)-treated rats as an inflammatory animal model to find that nicotine suppressed LPS-induced placental inflammation by inhibition of cytokine release and infiltration of leukocytes into the placenta through the cholinergic anti-inflammatory pathway [19]. Furthermore, we observed decreasing pup weights with LPS treatment and increasing pup weights with nicotine pretreatment in rats [20]. Here we aim to further investigate if activation of the cholinergic anti-inflammatory pathway by nicotine could protect fetus against LPS-induced FGR and further explore its possible mechanisms associated with placenta through pathological analysis and other quantitative method.

## Materials and methods

### Animals

The present study was carried out in the Laboratory Animal Center of Guangzhou Medical University in strict accordance with the recommendations in the Guide for the Care and Use of Laboratory Animals. The protocol was approved by the Committee on the Ethics of Animal Experiments of the Guangzhou Medical University (Permit Number: 2014-028). Sprague Dawley rats (200–240 g, 8–10 weeks) were purchased from the Medical Experimental Animal Center of Guangdong, China. Rats were housed under controlled laboratory conditions (temperature 24 ± 1°C, relative humidity 50–60%, light between 7:00 a.m. and 7:00 p.m.), with free access to standard rat chow and water, and were acclimated to light/dark cycle for at least 1 week before the experiment. Female rats were mated overnight with male rats. A positive sperm in vaginal smear after mating was designated as gestation day (GD) 1 of pregnancy (GD1).

### Reagents

LPS and nicotine (nicotine hydrogen tartrate salt) were both obtained from Sigma–Aldrich (St. Louis, MO, U.S.A.). Luminex kit was obtained from Bio-Rad (CA, U.S.A.).

#### Animal treatments

Pregnant Sprague–Dawley rats were divided into four groups (*n*=6/group) as follows: Group 1 rats (controls) were injected with vehicle (0.3 ml 0.9% NaCl, s.c.) on GD14 and GD15, and vehicle i.p. on GD16; Group 2 rats received an injection of LPS (25 μg/kg in 0.3 ml saline, i.p.) on GD16; Group 3 rats were first pretreated with nicotine (1 mg/kg/d, s.c.) on GD14 and GD15, then followed by LPS injection on GD16; and Group 4 rats were treated with nicotine (1 mg/kg/d in 0.3 ml saline, s.c.) alone on GD14 and GD15 [20]. All rats were killed on GD18.

#### Data and sample collection

All rats were killed on GD18. Number and weights of live fetuses were recorded. Similarly, number and weights of placentas were also recorded. Fetal loss (%) was estimated by dividing the empty fetal attachment sites, due to both absorption and early fetal expulsion, by total number of placentas with fetuses + empty placental attachment sites. Placentas in different groups were obtained for the subsequent experiments.

### Placental pathology

Placentas were collected, dissected longitudinally through the center, and one half fixed for 48 h in 10% formalin in PBS 0.1 M (pH 7.4), then dehydrated and embedded in paraffin. Longitudinal sections of 4 μm were made by a microtome (Leica RM 2125, Germany), mounted on slides, and stained with Hematoxylin and Eosin (H&E) and observed by light microscope (Leica DM2500, Germany).

### Luminex assay

Placentas were collected as described above. Total lysates were prepared by homogenizing 60 mg of placental tissue in 800 μl of RIPA lysis buffer. Placental IL-1, IL-6, TNF-α and vascular endothelial growth factor (VEGF) levels in the different groups were detected using Luminex 200 system and Bioplex HTF (Bio-Rad, CA, U.S.A.) in accordance with the manufacturer’s instructions. Standards and each sample were analyzed in duplicate. Data analysis was performed using Bio-Plex Manager, version 5.0 (Bio-Rad) and presented as concentrations (pg/ml/mg).

### Statistical analysis

All data were expressed as mean ± standard error of the mean (SEM) and statistical significance was accepted when *P*<0.05. Data analysis between multiple groups was performed using One-way analysis of variance (ANOVA) followed by the least significant difference (LSD) post hoc test or Dunnett’s test as appropriate. Power analyses (with at least an α of 0.05 and power of 0.80) were calculated for all statistical comparisons to assure that sufficient numbers of samples were used for measurements.

## Results

### Effects of LPS and nicotine on number of live fetuses and fetal loss

**Figure 1:**
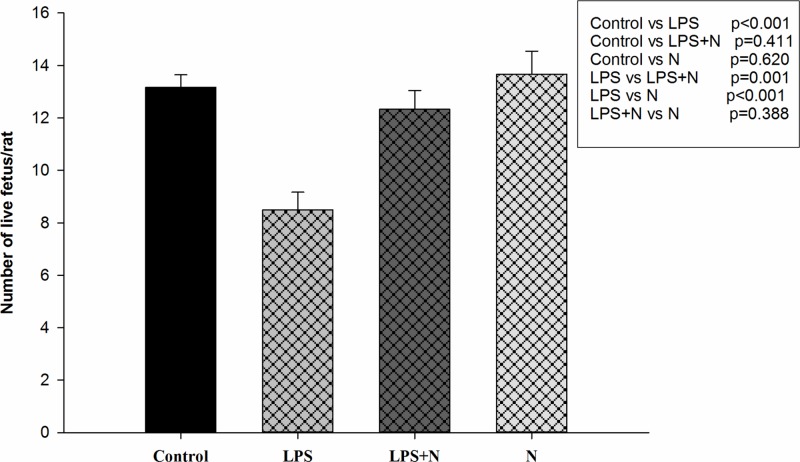
Number of live fetus/litter on GD18 after various treatments Bars represent means ± SEM of the live fetus number of six rats per group. Treatment with LPS alone significantly decreased the live fetus number compared with normal pregnant control (*P*<0.001), whereas treatment with LPS + nicotine significantly increased it compared with LPS alone (*P*=0.001). Significant differences were found between all groups as shown by *P*-values in the box inside the figure. Abbreviation: N, nicotine.

A similar number of live fetuses existed in control, LPS + nicotine and nicotine groups (13.17 ± 0.48, 12.33 ± 0.71 and 13.67 ± 0.88 live fetuses/litter, respectively, *P*>0.05). Treatment with LPS alone significantly decreased number of live fetuses on GD18 compared with control group (8.50 ± 0.67 vs 13.17 ± 0.48, *P*<0.001), while treatment with LPS + nicotine significantly increased number of live fetuses compared with LPS group (12.33 ± 0.71 vs 8.50 ± 0.67, *P*=0.001). LPS treatment significantly increased fetal losses (*P*<0.001), while treatment with LPS + nicotine significantly decreased fetal losses (*P*<0.001) (Table 1 and Figure 1).

**Table 1:** Fetal outcomes in various groups

Group	Live fetuses per litter (*n*)	Fetal loss (% of total)	Fetal weight on GD18 (g)	Placental weight on GD18 (g)
Control	13.17 ± 0.48^1^	3.65% (3/82)^1^	1.13 ± 0.01^1^	0.41 ± 0.06^1^
LPS	8.50 ± 0.67^2^	23.19% (16/69)^2^	0.90 ± 0.01^2^	0.38 ± 0.06^2^
LPS+Nicotine	12.33 ± 0.71^1^	10.98% (9/82)^3^	1.05 ± 0.01^3^	0.39 ± 0.05^2^
Nicotine	13.67 ± 0.88^1^	2.38% (2/84)^1^	1.11 ± 0.01^1^	0.40 ± 0.05^1^

Data are means ± SEM (*n*=6/group) of live fetuses per litter, fetal loss, and fetal and placental weights observed on GD18. LPS treatment deteriorated fetal outcomes compared with control group. Pretreatment of nicotine (LPS + nicotine) improved fetal outcomes compared with LPS group. There were significant differences in the live fetuses number per litter, fetal losses, fetal weights between LPS group and LPS + Nicotine group (*P*<0.05). However, difference in the placental weights in LPS group and LPS + Nicotine group were not significant (*P*>0.05). Different superscripts after means and SEM of each column (i.e., Live fetuses per litter, Fetal loss, Fetal weight on GD18 and placental weight on GD18) indicate significant differences (*P*<0.05) between groups.

### Nicotine ameliorates LPS-induced FGR on GD18

**Figure 2:**
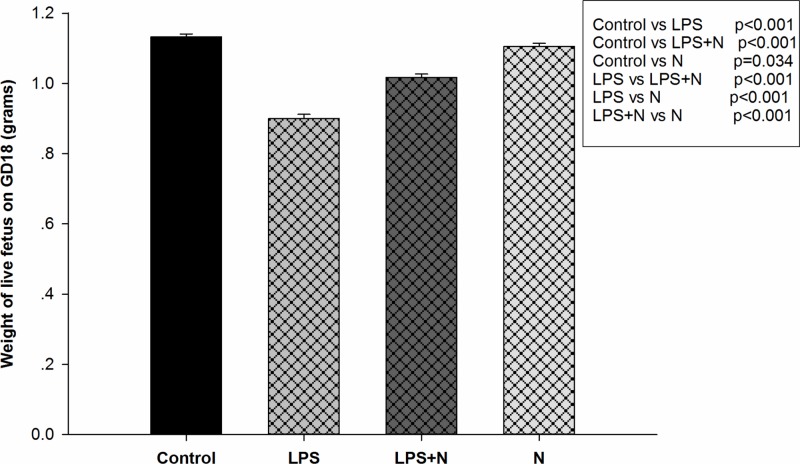
Weights of live fetuses on GD18 after various treatments Bars represent means ± SEM of the live fetus weight of six rats per group. Treatment with LPS alone significantly reduced live fetus weight on GD18 (*P*<0.001), whereas treatment with LPS + nicotine significantly increased live fetus weight on GD18 compared with LPS alone (*P*<0.001) (data as shown in Figure 1).

Live fetus weight in LPS group was more than 10th percentile lower than that in control group on GD18 (0.90 ± 0.01 vs 1.13 ± 0.01 g, *P*<0.001), which meant treatment with LPS alone markedly reduced live fetus weight and induced FGR in pregnant rats. Pretreatment with nicotine (LPS + nicotine) significantly alleviated an LPS-induced decrease in fetal weight and protected fetus against LPS-induced FGR (1.02 ± 0.01 vs 0.90 ± 0.01 g, *P*<0.001). No significant difference was found between control group and nicotine group (*P*=0.034) (Table 1 and Figure 2).

### Effects of LPS and nicotine on placental weight and placental pathology

**Figure 3:**
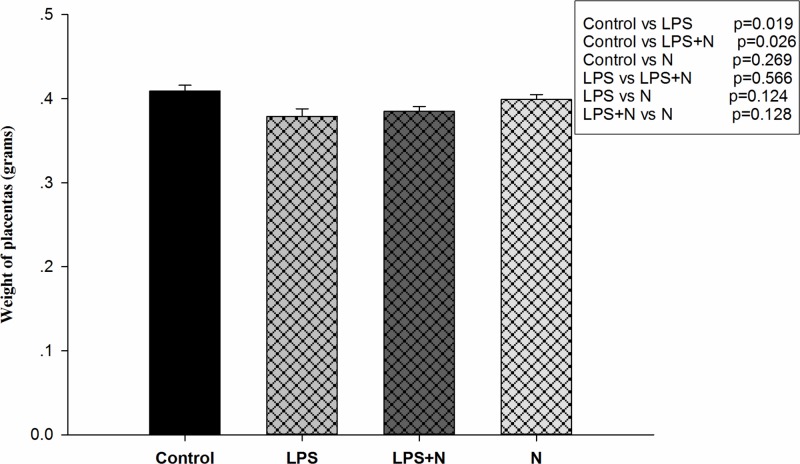
Weights of placentas on GD18 in different groups Bars represent means ± SEM of weight of placentas. Weights of placentas in LPS group and LPS + nicotine group were lower than those in control group (p = 0.019, p = 0.026, respectively). No significant difference was found between nicotine group and control group (p = 0.269), and between LPS + nicotine group and LPS group (p = 0.566).

The LPS and LPS + nicotine treatments decreased placental weights compared with control group (*P*=0.019, *P*=0.026, respectively). There was no significant difference in the placental weights between nicotine group and control group (*P*=0.269), and between LPS + nicotine group and LPS group (Table 1 and Figure 3) (*P*=0.566).

**Figure 4:**
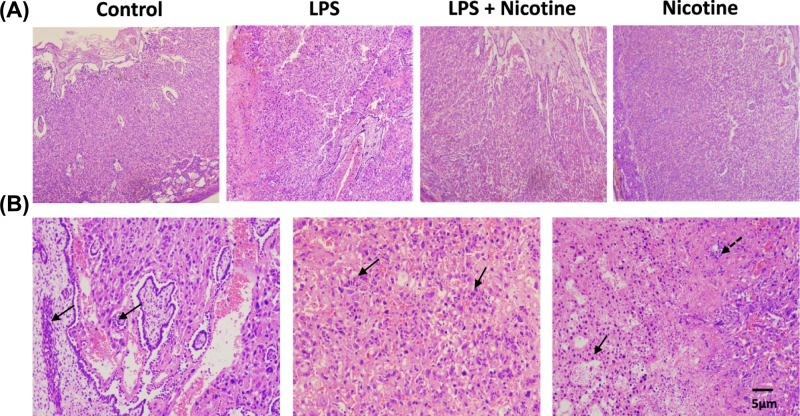
Nicotine suppressed LPS-induced placental pathological changes (**A**) Representative photomicrographs by HE staining of placentas from different groups (40×). (**B**) HE staining of placentas from LPS group (100×). **Left:** space of intervillous and minute vessels decreased in villi, solid arrows indicate inflammatory cells infiltrated in dysontogenesis villi; **Middle:** solid arrows indicate inflammatory cells infiltrated in decidua; **Right:** solid arrow indicates LPS-induced abscess in fetal membrane, dashed arrow indicates LPS-induced necrosis in fetal membrane.

Obvious pathological changes were observed in placentas of LPS-treated rats: inflammatory cells infiltrated in dysontogenesis villi and decidua, space of intervillous and minute vessels in villi deceased, abscess and necrosis of fetal membrane (Figure 4B). These changes in LPS group always accompanied with decreasing placental weight and fetal weight. However, these pathological changes were not observed in placentas of nicotine pretreatment group (LPS + nicotine), control group and nicotine group. The placentas in these groups were characterized with normal placental structure and few inflammatory cells (Figure 4A).

### Effects of LPS and nicotine on levels of placental inflammatory cytokines and VEGF

**Figure 5:**
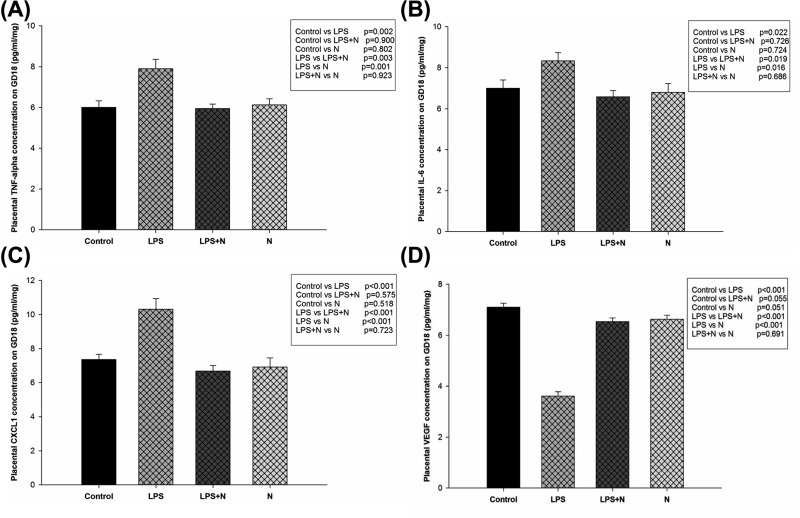
Placental TNF-α, IL-6, CXCL1 and VEGF levels on GD18 in different groups Bars represent means ± SEM of concentrations (pg/ml/mg). (**A**–**C**) There were increased TNF-α, IL-6 and CXCL1 levels in the LPS treatment group compared with the control group (*P*<0.001), and decreased TNF-α, IL-6 and CXCL1 levels in the LPS + nicotine group (LPS + N) and nicotine group compared with LPS group (*P*<0.001). (**D**) There were decreased VEGF levels in the LPS treatment group compared with the control group (*P*<0.001), and increased VEGF levels in the LPS + nicotine group (LPS + N) and nicotine group compared with LPS group (*P*<0.001).

LPS-treated rats with serious placental inflammation demonstrated increase in the placental levels of TNF-α, IL-6, and CXCL1 levels and decrease in VEGF levels compared with control group (7.91 ± 0.45 vs 6.01 ± 0.32, 8.34 ± 0.39 vs 7.00 ± 0.41, 10.31 ± 0.63 vs 7.36 ± 0.31, 3.61 ± 0.18 vs 7.10 ± 0.16, pg/ml/mg, respectively, *P*<0.05). Treatment with LPS + nicotine significantly decreased placental TNF-α, IL-6, CXCL1 levels and increased VEGF levels compared with LPS alone (5.95 ± 0.22 vs 7.91 ± 0.45, 6.58 ± 0.30 vs 8.34 ± 0.39, 6.68 ± 0.33 vs 10.31 ± 0.63, 6.53 ± 0.15 vs 3.61 ± 0.18, pg/ml/mg, respectively, *P*<0.05), accompanied with normal histomorphological characteristics of placentas. Nicotine treatment alone had no effect on the placental cytokines and VEGF levels compared with controls (*P*>0.05) (Figure 5).

## Discussion

In the current study, we investigated that activation of the cholinergic anti-inflammatory pathway by nicotine might protect fetus against LPS-induced FGR through ameliorating placental inflammation and vascular development in late gestation in pregnant rats. We found that pretreatment of nicotine on GD14 and GD15 could prevent LPS-induced increase in placental TNF-α, IL-6, CXCL1 levels, decrease in VEGF levels, placental pathological injury and fetal losses, and could increase weights of live fetuses on GD18.

As it is well known, fetal development is totally reliant on the placenta, a transient extracorporeal organ that interfaces with the mother, to sustain and protect it. Fetal growth can only take place at a rate commensurate with that of the delivery of nutrients and oxygen by the placenta [21]. Impaired placental structure and function is a significant factor in fetal growth abnormalities, including FGR, fetal loss and other complications [22–25]. We previously observed placentas with more inflammatory cells infiltrating and more inflammatory cytokines production after LPS treatment in pregnant rats and suggested that nicotine might exert its anti-inflammatory role (inhibiting LPS-induced cytokines production and leukocytes infiltration) via combination with the α7-nAChR, which expressed in rat placenta [19,20,26]. However, it was not confirmed whether FGR existed in late gestation of these LPS-treated pregnant rats. Moreover, the effects of LPS and nicotine on placental weight and pathology, and the possible protective effects of nicotine on fetus in late gestation of rats were not evaluated.

In the present study, intraperitoneal injection of LPS on GD16 in pregnant Sprague–Dawley rats, significantly decreased weights of live fetuses (more than 10th percentile lower) on GD18, and induced FGR, accompanied with increased fetal losses and decreased number of live fetuses. This is consistent with what we often encounter in clinical practice, as FGR is always associated with a high incidence of perinatal morbidity and mortality [24,27]. Similar LPS-induced FGR models have been applied to other relevant studies. Furthermore, pretreatment of nicotine by subcutaneous injection on GD14 and GD15 significantly rescued weights of live fetuses, accompanied with decreased fetal losses and increased number of live fetuses. These data demonstrated the protective effects of nicotine on LPS-induced FGR.

Since placenta is an important organ supplying the fetus, we extended these observations and investigated weights and histomorphological characteristics of placentas in the different groups.

Decreased placental weights and obvious placental pathological changes were found in LPS-treated rats. Placental pathological changes included abscess and necrosis of fetal membrane, infiltration of inflammatory cells in dysontogenesis villi and decidua, decreasing intervillous space and minute vessel in villi. These reduced valid villous surface area and decreased terminal villous capillary number undoubtedly affected the normal nutrients and oxygen supply from mother to the fetus [28].

Further, we quantitatively determined the placental inflammatory cytokines and VEGF levels in different groups. LPS treatment increased the placental levels of TNF-α, IL-6, CXCL1 *in vivo* as we reported in our previous *in vitro* studies [19], which suggested serious placental inflammation. These cytokines were involved in breaking the normal structure of placentas, and in turn, the damaged cells in placentas released more inflammatory cytokines and other mediators to initiate inflammatory cascades, for example recruiting more neutrophils into the chorionic plate and decidua [29], reducing VEGF production and decreasing minute vessels in villi. VEGF might play an important protective role against LPS-induced placental vascular injury as a regulator in the growth and development of blood vessels [30], and pretreatment of nicotine might protect fetus through restoring placental VEGF production and promoting placental vascular development damaged by LPS, in addition to suppressing placental inflammation.

Combining the results of our previous studies, current studies and other relevant studies, we speculated that administration of LPS activated maternal inflammatory response, increased inflammatory cytokines and leukocytes infiltration in placenta, increased the production of downstream mediators, and destroyed placental structure and function, and further resulted in decreased VEGF, impaired vascular development in villi, and insufficient oxygen and nutrients supply to fetus and eventually led to FGR. Nicotine, as an α7-nAChR agonist, activated cholinergic anti-inflammatory pathway, suppressed inflammatory cytokines release and leukocytes infiltration into the placenta, reversed the placental injuries from LPS treatment, restored normal VEGF production and placental vascular development, and then protected fetus against FGR. However, it seemed that nicotine failed to rescue placental weight on GD18 in our study. This might be attributable to some irreversible LPS-induced injuries on placenta or the very small change of placental weight.

The present study clearly showed that activation of the cholinergic anti-inflammatory pathway by nicotine protected fetus against LPS-induced FGR through ameliorating placental inflammation and vascular development in late gestation in pregnant rats. It is consistent with other studies which also demonstrated the effects of nicotine or other agonists on promoting fetal growth [20,31,32]. However, there were still other studies which reported low birth weight and organ damage as adverse effects of nicotine exposure during pregnancy [33–37]. These studies were different in the duration and dose of nicotine exposure compared with our study. In our study, nicotine was injected subcutaneously only on GD14 and GD15 (1 mg/kg/day), but in the other studies, nicotine was either administrated pre-pregnancy, during all of pregnancy or for at least 1 week in pregnancy (1–6 mg/kg/day). It is also worth mentioning that one of our studies suggested choline’s oral administration maybe an alternative and more acceptable way to apply, since choline is both a natural agonist of α7-nAChR and an essential nutrient that functions in phospholipid metabolism and neurotransmission and as a methyl donor [31,32,38]. However, further studies are needed to evaluate the side effects of nicotine on fetuses.

In summary, our study revealed that nicotine and possibly other α7-nAChR agonists might help to protect fetus against LPS-induced FGR in late pregnancy in rats through ameliorating placental inflammation and vascular development. Further additional studies would be useful to explore the potential strategies of treatments with α7-nAChR agonists or some cytokines against FGR.

## Abbreviations

CXCL1: chemokine (C-X-C motif) ligand 1
FGR: fetal growth restriction
GD: gestation day
HE: hematoxylin-eosin
IL: interleukin
i.p.: intraperitoneal
LPS: lipopolysaccharide
RIPA: radio-immunoprecipitation assay
s.c.: subcutaneous
VEGF: vascular endothelial growth factor
α7-nAChR: α7 nicotinic acetylcholine receptor
